# Oral administration of live- or heat-killed *Candida albicans* worsened cecal ligation and puncture sepsis in a murine model possibly due to an increased serum (1→3)-β-D-glucan

**DOI:** 10.1371/journal.pone.0181439

**Published:** 2017-07-27

**Authors:** Wimonrat Panpetch, Naraporn Somboonna, Dewi Embong Bulan, Jiraphorn Issara-Amphorn, Malcolm Finkelman, Navaporn Worasilchai, Ariya Chindamporn, Tanapat Palaga, Somying Tumwasorn, Asada Leelahavanichkul

**Affiliations:** 1 Interdisciplinary Program of Medical Microbiology, Graduate school, Chulalongkorn University, Bangkok, Thailand; 2 Department of Microbiology, Faculty of Medicine, Chulalongkorn University, Bangkok, Thailand; 3 Department of Microbiology, Faculty of Science, Chulalongkorn University, Bangkok, Thailand; 4 Omics Sciences and Bioinformatics Center, Faculty of Science, Chulalongkorn University, Bangkok, Thailand; 5 Department of Water Resources Management, Faculty of Fisheries and Marine Science, Mulawarman University, Indonesia; 6 Associates of Cape Cod, Inc., East Falmouth, Massachusetts, United States of America; 7 Center of Excellence in Immunology and Immune-mediated Diseases, Department of Microbiology, Faculty of Medicine, Bangkok, Thailand; 8 STAR on Craniofacial and Skeleton Disorders, Faculty of Dentistry, Chulalongkorn University, Bangkok, Thailand; Louisiana State University, UNITED STATES

## Abstract

*Candida albicans* is the most common fungus in the human intestinal microbiota but not in mice. To make a murine sepsis model more closely resemble human sepsis and to explore the role of intestinal *C*. *albicans*, in the absence of candidemia, in bacterial sepsis, live- or heat-killed *C*. *albicans* was orally administered to mice at 3h prior to cecal ligation and puncture (CLP). A higher mortality rate of CLP was demonstrated with *Candida*-administration (live- or heat-killed) prior to CLP. Fecal *Candida* presented only in experiments with live-*Candida* administration. Despite the absence of candidemia, serum (1→3)-β-D-glucan (BG) was higher in CLP with *Candida*-administration than CLP-controls (normal saline administration) at 6h and/or 18h post-CLP. Interestingly, fluconazole attenuated the fecal *Candida* burden and improved survival in mice with live-*Candida* administration, but not CLP-control. Microbiota analysis revealed increased *Bacteroides* spp. and reduced *Lactobacillus* spp. in feces after *Candida* administration. Additionally, synergy in the elicitation of cytokine production from bone marrow-derived macrophages, *in vitro*, was demonstrated by co-exposure to heat-killed *E*. *coli* and BG. In conclusion, intestinal abundance of fungi and/or fungal-molecules was associated with increased bacterial sepsis-severity, perhaps through enhanced cytokine elicitation induced by synergistic responses to molecules from gut-derived bacteria and fungi. Conversely, reducing intestinal fungal burdens decreased serum BG and attenuated sepsis in our model.

## Introduction

Sepsis is a syndrome of imbalance of host pro- and anti-inflammatory responses to pathogens [1, 2]. Sepsis is a critically important worldwide health-care problem and is the most common cause of death among patients in the intensive care unit [3, 4]. Pathogen associated molecular patterns (PAMPs) derived from gastrointestinal (GI) microorganisms are capable of immune activation and gut-translocation of viable bacteria or bacterial molecules leads to vigorous systemic inflammation [5]. Indeed, gut permeability barrier defects are observed in sepsis [6, 7]. While the significance of gut-translocation of bacterial molecules is appreciated [8], the impact of fungal molecules in bacterial sepsis is unknown.

(1→3)-β-D-glucan (BG) are a major component of the cell wall in most fungi and are released during fungal-growth and the tissue invasion process [9, 10]. BG are bioactive and activate immune responses through several receptors [11, 12]. We have demonstrated that in bacterial sepsis, higher serum BG, from gut-translocation, in the absence of fungemia, is associated with greater sepsis severity [7]. However, the role of intestinal fungi in bacterial sepsis in the absence of fungemia is not well studied. In order to address the role of fungi, we assessed the effect of oral administration of *C*. *albicans* in a murine bacterial sepsis model. Because *C*. *albicans* is the predominant fungal species in human intestine but not in mouse [13], a murine sepsis model with orally-administered *C*. *albicans* might more closely resemble human sepsis. We recently demonstrated that oral administration of *C*. *albicans* with mixed-oral antibiotics 5 days prior to cecal ligation and puncture (CLP) enhances the severity of bacterial sepsis in the murine sepsis model [14]. However, oral antibiotics, alone, impact fecal microbiota and sepsis severity in the *Candida* colonization model [14]. Hence, the influence of *C*. *albicans* in bacterial sepsis might be demonstrated more accurately without antibiotic administration.

Accordingly, the importance of intestinal fungi, without fungemia, in bacterial sepsis was investigated using a murine cecal ligation and puncture (CLP) sepsis model with *C*. *albicans* administered orally at 3h, but not 5 days, prior to the surgery without oral antibiotics administration.

## Materials and methods

### *Candida albicans* preparation

Fluconazole-sensitive *Candida albicans* ATCC 90028 (Fisher Scientific, Waltham, MA, USA; minimal inhibitory concentration: 0.25–1 μg/ml) was used. *C*. *albicans* were cultured over-night on Sabouraud dextrose broth (SDB) (Thermo Scientific, Hampshire, UK) and counted in a hemocytometer (Bright-Line, Denver, CO, USA) before use. Heat-killed *C*. *albicans* were prepared by immersion in a water-bath at 60°C for 1h.

### Animals and animal models

The US National Institutes of Health (NIH) animal care and use protocol (#85–23, revised 1985) was followed. Male, ICR mice at 8-week-old (National Laboratory Animal Center, Nakhornpathom, Thailand) were used. The animal protocols were approved by the Institutional Animal Care and Use Committee of the Faculty of Medicine, Chulalongkorn University, Bangkok, Thailand.

### Cecal ligation and puncture at 3h after oral-administration of *Candida albicans* (CLP with immediate *Candida* administration model)

Live- *C*. *albicans* oral administration at 1x10^6^ CFU, with cecal ligation and puncture (CLP) surgery, induced positive fecal fungi without fungemia, within 24h after administration. Oral *Candida* at higher doses, with CLP, induced positive fungal growth from both feces and blood. Live- or heat-killed *C*. *albicans* at 1x10^6^ CFU was administered at 3h prior to cecal ligation and puncture (CLP) surgery to characterize the potential role of (1→3)-β-D-glucan (BG) in bacterial sepsis. CLP procedures were slightly modified from the previously published [7]. Briefly, the cecum was ligated at 10 mm from the cecal tip and punctured twice with a 21-gauge needle. The operation was performed through an abdominal incision under isoflurane anesthesia. Fentanyl at 0.03 mg/kg in 0.5 ml of normal saline solution (NSS) was administered subcutaneously for an analgesia and fluid replacement post-operatively and at 6h. Antibiotic (imipenem/cilastatin) 14 mg/kg in 0.3 ml of NSS was administered subcutaneously at 6h post-surgery. Fluconazole (Sigma-Aldrich, St. Louis, MO, USA) at 10 mg/kg in 0.5 ml of NSS or NSS alone was administered orally, pre-operatively (0.5h), and post-operatively at 3h and 6h to reduce fecal *Candida*. High dose fluconazole was needed to significantly reduce fecal *Candida* [15]. In the sham operation, cecum was only identified through the abdominal incision then sutured.

### Mouse sample analysis

Blood collection was performed at the indicated time-points by tail vein nicking and at sacrifice with cardiac puncture under isoflurane anesthesia. For blood bacterial quantitative analysis, blood (25 μl) was spread directly onto blood agar plates (Oxoid, Hampshire, UK), incubated at 37°C and bacterial colonies were enumerated at 24-48h. Serum was separated by centrifugation and kept at -80°C until analysis. The QuantiChrom Creatinine Assay kit (DICT-500; Bioassay, Hayward, CA, USA) and EnzyChrom Alanine Transaminase assay (EALT-100, BioAssay) were used for serum creatinine (Scr) and alanine transaminase (ALT) measurement, respectively. Serum cytokines (TNF-α, IL-6 and IL-10) were measured with ELISA (ReproTech, NJ, USA). BG was analyzed with Fungitell^®^ (Associates of Cape Cod, Inc., East Falmouth, MA). BG values at <7.8 and >523.4 pg/ml (beyond the lower and upper range of the standard curve) were recorded as 0 and 523 pg/ml, respectively. All assays were performed according to the manufacturer’s protocol. In addition, at 18h post-CLP, internal organs; liver, spleen, and mesenteric lymph nodes were weighed, homogenized and plated onto blood agar plates and processed as previously mentioned.

### Culture of fungi

Individual mice were placed in an empty cage prior to CLP for fecal collection (0h time-point of CLP). At 6h and 18h post-CLP, mice were sacrificed and feces from descending colon and/or rectum were collected. Feces and phosphate buffer solution (PBS) in a ratio of 1μg/ 1μl were well-mixed before plating directly onto Sabouraud dextrose agar (SDA) with 0.1% chloramphenicol (Thermo Scientific). The plates were incubated at 35°C, for 72h, before fungal colony enumeration. In addition, at 18h post-CLP, internal organs; liver, spleen, and mesenteric lymph nodes were weighed, homogenized and plated onto SDA with 0.1% chloramphenicol and processed as previously mentioned.

### Fecal microbiome

Microbiota analysis, as previously reported [16], was used to explore the alteration of fecal microbiota in each manipulation. Briefly, feces from individual mice (0.25g) were processed for metagenomic DNA extractions. 3 independent extractions were performed per sample. Total nucleic acid was extracted by power DNA Isolation Kit (MoBio, Carlsbad, CA, USA). Agarose gel electrophoresis and nanodrop spectrophotometry were used to assess metagenomic DNA quality. Universal prokaryotic 515F (forward; (5′-GTGCCAGCMGCCGCGGTAA-3′) and 806R (reverse; 5′-GGACTACHVGGGTWTCTAAT-3′), with appended 5′ Illumina adapter and 3′ Golay barcode sequences, were used for 16S rRNA gene V4 library construction [16]. Each 25-μl PCR reaction comprised 1× EmeraldAmp^®^ GT PCR Master Mix (TaKaRa), 0.2 μM of each primer, and 75 ng of the metagenomic DNA. Independently triplicate polymerase chain reactions (PCRs) were performed and pooled to prevent stochastic PCR bias. The 16S rDNA amplicons of 381 basepairs (bp) were purified from agarose gel, using the GenepHlow^™^ Gel Extraction Kit (Geneaid Biotech Ltd., New Taipei City, Taiwan), and quantified by Picogreen (Invitrogen, Eugene, Oregon, USA). Each sample (240 ng) was pooled for Miseq300 platform sequencing (Illumina, San Diego, CA, USA), by the sequencing primers and index sequence described [17].

Mothur’s standard quality screening operating procedures for MiSeq platform was used for the quality screening of the raw sequences [18] then aligned and assigned taxon (operational taxonomic unit, OTU) based on a default parameter [18]. Samples were normalized to an equal sampling depth (N = 118121 reads per sample) followed Mothur’s computations method [18].

### Bone marrow derived macrophage preparation

A previous published method of preparing bone marrow (BM) derived-macrophages was used [19]. In brief, femur BM cells were incubated for 7 days in supplemented Dulbecco's Modified Eagle Medium (DMEM) with 20% conditioned medium of the L929 cell line, as a source of macrophage-colony stimulating factor (M-CSF), in a humidified 5% CO_2_ incubator at 37°C. Cells were harvested with cold PBS. Anti-F4/80 and anti-CD11c antibody staining (BioLegend, San Diego, CA, USA) by flow cytometry were used for characterization of the macrophage phenotype.

### Induction of macrophage cytokine production

The preparation of heat-killed *E*. *coli* (*Escherichia coli* ATCC 25922; ATCC, Manassas, VA, USA) was used as representative of bacterial molecules. Bacteria (3.3x10^7^ cells/ ml) were killed by heating at 65°C for 15 minutes followed by sonication with a High Intensity Ultrasonic Processor (VC/VCX 130, 500,750) at 25 percent amplitude until a clear solution was obtained. The lysate was centrifuged at 10,000 rpm for 5 minutes, and the supernatant was decanted and filtered with 0.22 μm filters (Millex^®^, Tullagreen, Carrigtwohill, Ireland). For a representative BG, CM-Pachyman, (Megazyme, Bray, Ireland) was used. [20]. Pachyman at different doses, with or without *E*. *coli* preparations, were incubated with macrophages (1x10^5^ cells/well) in culture plates. The total volume was adjusted by PBS addition to 200 μl/well. Supernatant was collected at specific time-points and cytokines were measured by ELISA assay (ReproTech).

### Statistical analysis

Data was presented as mean ±standard error (SE) and the differences between groups were examined for statistical significance by one-way analysis of variance (ANOVA) followed by Tukey’s analysis or the Mann-Whitney U test for the comparisons of multiple or 2 groups, respectively. Survival analysis was performed by log-rank test. The repeated measures analysis of variance (ANOVA) with Bonferroni post hoc analysis was used for the analysis of the time-course experiments. All statistical analyses were performed with SPSS 11.5 software (SPSS, IL, USA). In addition, the area under the curve (AUC) of cytokine response was calculated with Graphpad Prism4.0 software and compared between the mouse strains with one-way ANOVA followed by Tukey’s analysis (SPSS). A *P* value < 0.05 was considered to be statistically significant.

## Results

### Oral administration of live- or heat-killed- *Candida albicans* before cecal ligation and puncture increased serum (1→3)-β-D-glucan and worsened sepsis severity

Without *Candida* spp. administration, fecal fungi were undetectable, with, or without, CLP (data not shown), as observed previously [13, 21, 22]. Oral administration of live *Candida* at 10^4^, 10^6^ and 10^10^ CFU/mouse alone, without CLP, did not cause candidemia. However, a low level of candidemia (70±34 CFU/ml; data from 8 mice) was demonstrated at 18h post-CLP with *Candida* gavage at 10^10^ CFU/mouse. Subsequently, live- or heat-killed- *Candida* at 10^6^ CFU, an optimal *Candida* dose which did not induce candidemia, was selected for the further studies.

**Fig 1:**
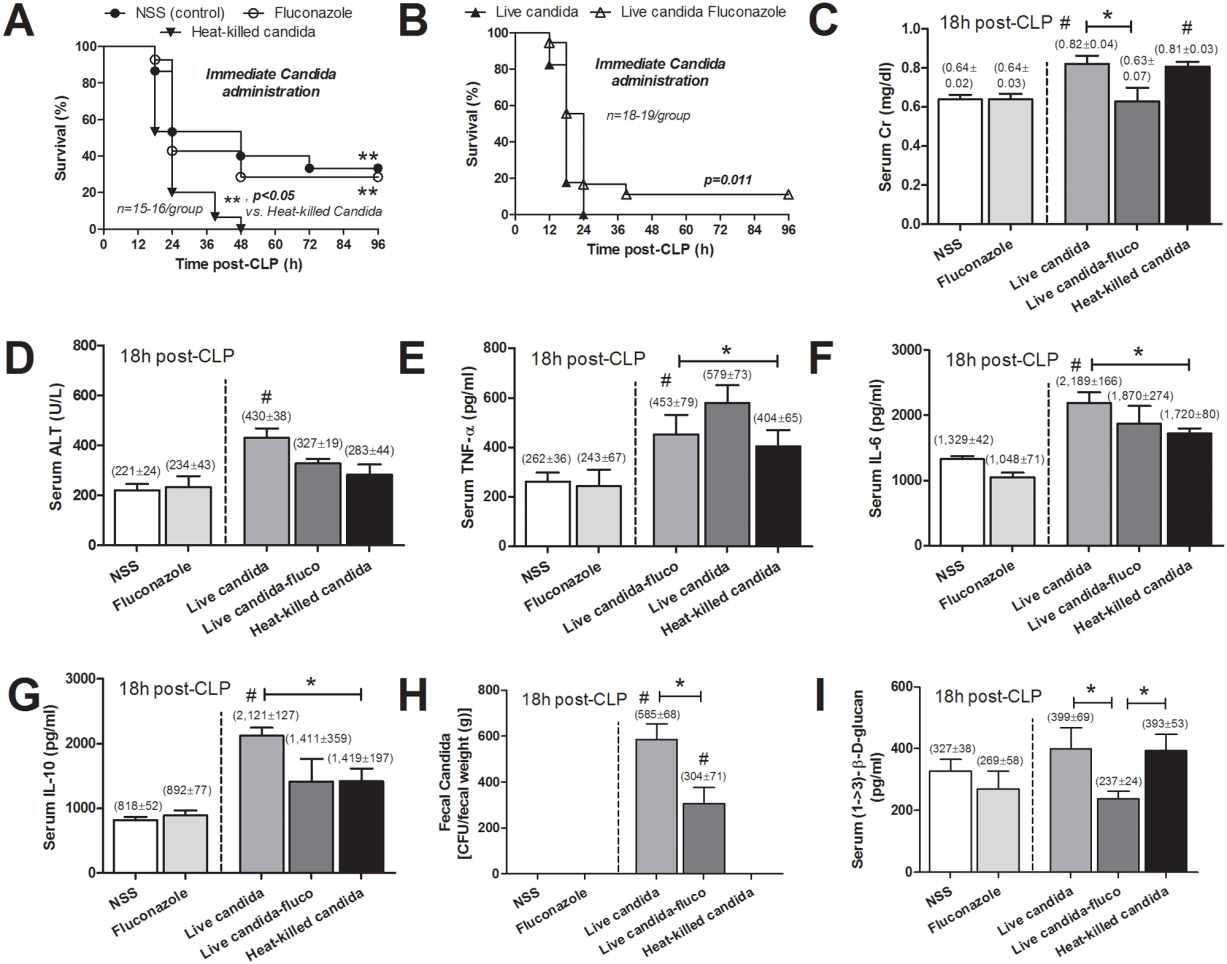
Survival analysis of mice with cecal ligation and puncture (CLP) with normal saline (NSS), NSS with fluconazole and heat-killed *Candida* oral administration (A) and with live-*Candida* with and without fluconazole (B) at the time of operation is shown. Organ injury, as demonstrated by serum creatinine (Scr) for kidney injury (C), alanine transaminase (ALT) for liver injury (D), serum cytokines (TNF-α, IL-6 and IL-10) (E-G), fecal *Candida* burdens (H) and serum (1→3)-β-D-glucan (BG) (I) was analyzed.(n = 6-7/group); #, *p* < 0.05 vs. NSS; *, *p* < 0.05

Live- or heat-killed *C*. *albicans* was orally administered at 3h before CLP to evaluate the effect of live- fungi or fungal molecules upon bacterial sepsis. The administration of either live- or heat-killed *C*. *albicans* increased sepsis mortality rate to 100% mortality within 48h after surgery in both models; compared with 67% in CLP with NSS control (CLP-control) (Fig 1A and 1B). In addition, enhanced sepsis severity was also demonstrated by Scr (kidney injury), ALT (liver injury) and cytokines (TNF-α, IL-6 and IL-10) at 18h post-CLP (Fig 1C–1G). All of these parameters, in CLP with live-*Candida* administration (CLP-live-*Candida*), were higher than CLP-control. In addition, fecal *Candida* was detectable only in CLP after live *Candida* administration and fecal *Candida* burdens were higher in CLP-live-*Candida* without fluconazole (Fig 1H). On the other hand, serum BG was higher in CLP-live-*Candida* with fluconazole compare to CLP with live- or heat-killed *Candida* (Fig 1I). This implied the enhanced serum BG after an oral BG administration.

**Fig 2:**
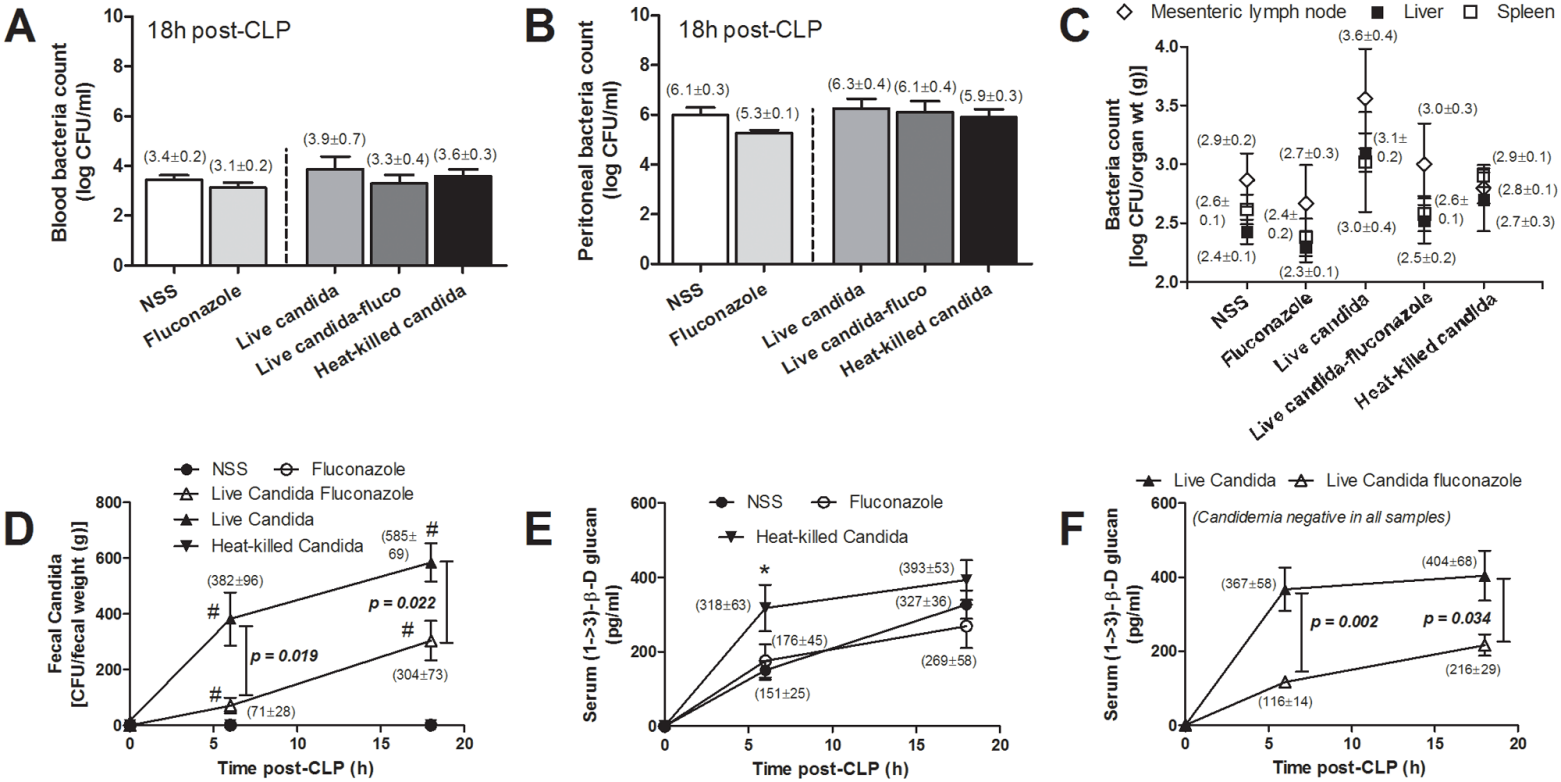
Blood bacterial count (A), peritoneal bacterial count (B) and bacterial count from internal organs (C) are shown. (n = 7/ group). The time-course of fecal *Candida* burdens (D) and serum (1→3)-β-D-glucan (BG) in different treatment groups (E, F) is described. [n (at 0h) = 10-12/group and n (at 6h and 18h) = 5-6/group]. #, *p* < 0.05 vs. 0h in each group; *, *p* < 0.05 vs. NSS

Moreover, Scr but not serum BG, ALT and cytokines in CLP with heat killed-*Candida* was higher than CLP-control (Fig 1C–1G). Serum cytokines, but not Scr and ALT, in CLP-live-*Candida* were higher than CLP with heat-killed-*Candida*. On the other hand, quantitative analysis of blood bacterial count, peritoneal bacterial count and bacterial count from the internal organs at 18h post-CLP were positive for mixed bacterial colonies but the levels were not different among groups (Fig 2A–2C).

**Fig 3:**
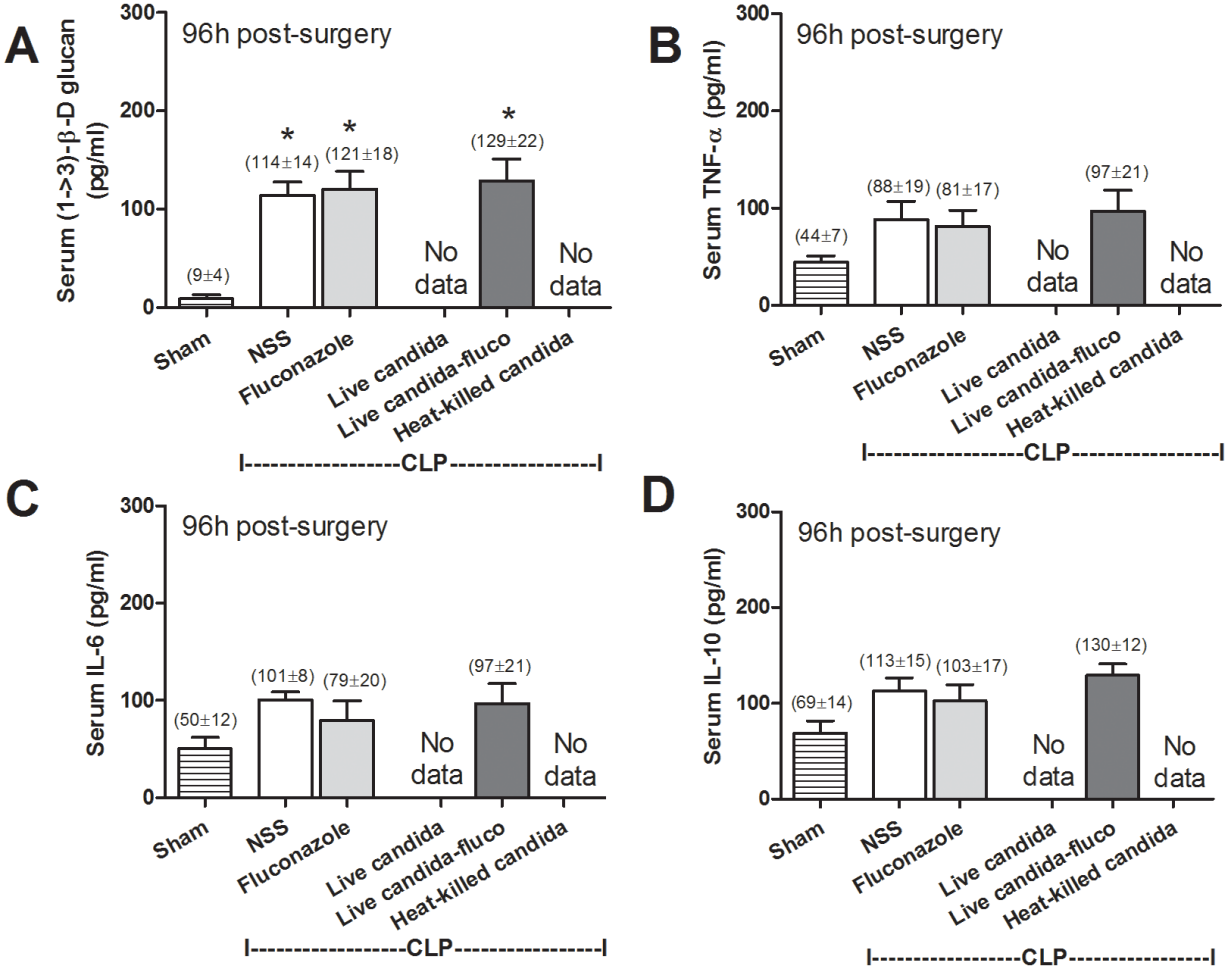
Serum (1→3)-β-D-glucan (BG) (A) and serum cytokines (TNF-α, IL-6 and IL-10) (B-D) in the mice at 96h post-sham surgery (n = 5) and mice survived at 96h post-CLP administered with normal saline (NSS; n = 5), fluconazole (n = 4) and live-*Candida* with fluconazole (n = 2) are shown (survival rate of these groups were demonstrated in Fig 1A and 1B). *, *p* < 0.05 vs. sham; No data, non survivor in this group.

Oral live-*Candida* administration converted fecal *Candida* culture from negative to positive in a time dependent manner after CLP despite the absence of candidemia (Fig 2D). In comparison with CLP-control, serum BG was higher in CLP with heat-killed *Candida* administration at 6h but not at 18h after CLP (Fig 2E). And serum BG was higher in CLP-live-*Candida* at 6h and 18h in comparison with CLP-control (Fig 2F). At 6h, serum BG of CLP-control, heat-killed- and live- *Candida* were 150±25, 318±63 and 367±58 pg/ml, respectively; *p*<0.05 by one-way ANOVA. Of note, fungal culture was negative in blood, peritoneal fluid and the internal organs (data not shown). Interestingly, fluconazole reduced intestinal *Candida* abundance (Fig 2D) and attenuated sepsis severity in CLP-live-*Candida* (Fig 1B). Survival in CLP with live-*Candida*, CLP-control and CLP-fluconazole were 2/18 (11%), 5/15 (33%) and 4/14 (29%), respectively (Fig 1A). In parallel, fluconazole in CLP-live-*Candida* attenuated fecal *Candida*, serum BG, mortality rate and Scr, but not ALT and serum cytokines (Fig 2D–2F, Fig 1). In contrast, fluconazole was non-effective for sepsis attenuation in CLP-control group (Figs 1 and 2). It is interesting to note that both serum BG and serum cytokine levels were lower in the survivors at 96h post-CLP in comparison with 18h-post CLP. However, while the serum cytokines were normalized, serum BG level was still higher than sham control (Fig 3).

### Oral administration of either live- or heat-killed-*Candida albicans* before cecal ligation and puncture increased several intestinal anaerobic bacteria but not aerobic bacteria

**Fig 4:**
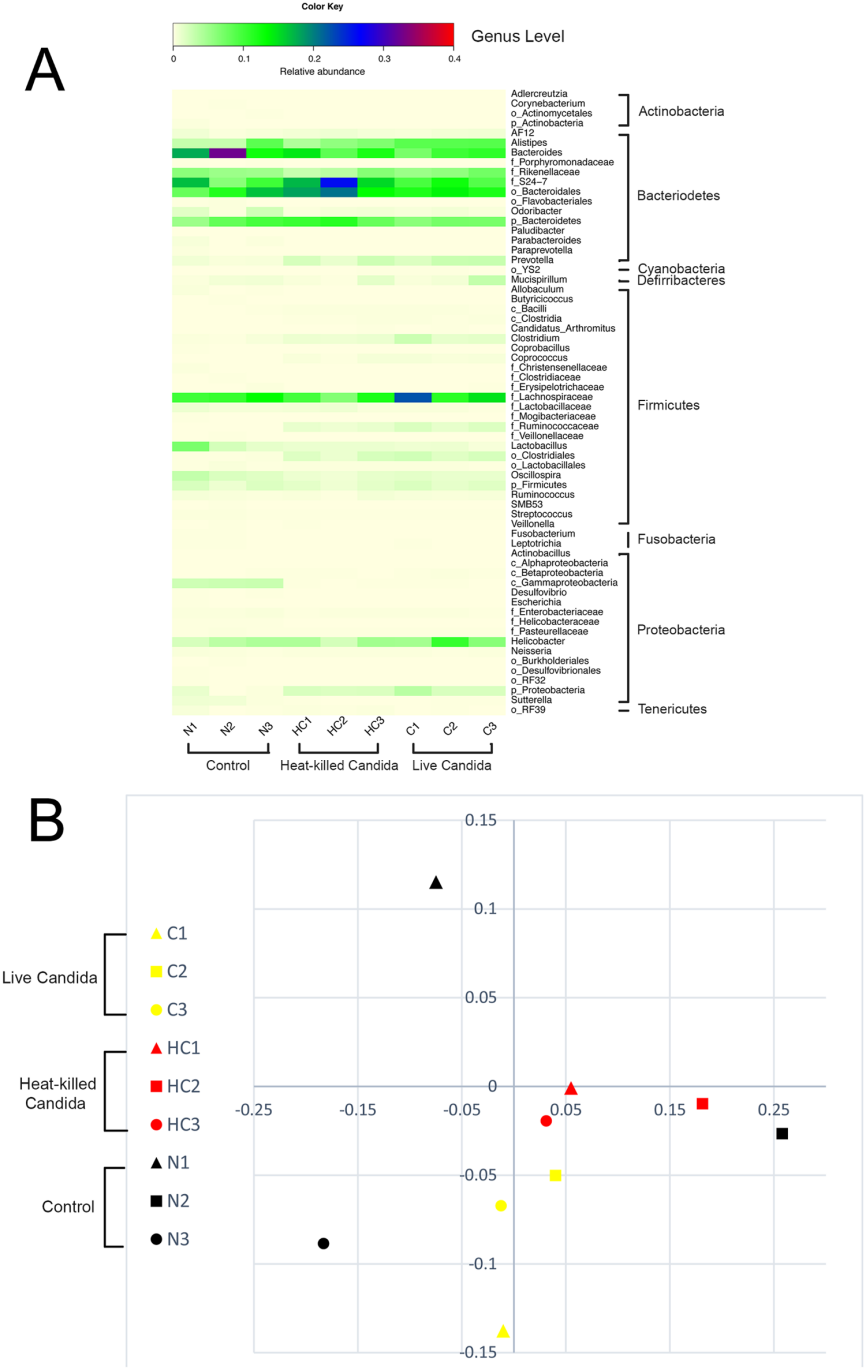
Gut microbiota analysis from individual mouse feces. Control (N1-3), heat-killed *Candida* administration (HC1-3) and live-*Candida* (C1-3) by relative abundance of bacterial diversity at genus level (A) are shown. Abbreviation “p”, “c” and “o” means unclassified family sequences in phylum, class and order, respectively, and Genera that contain ≤ 0.01% relative abundance were removed. A representative of gut microbiota structures demonstrated by non-metric multidimensional scaling (NMDS) are shown (B).

Because gram negative bacteria are well-known causative agents of severe bacterial sepsis, it is possible that oral *Candida* administration might enhance gram negative bacteria in gut. Accordingly, we characterized the fecal gut bacterial microbiota (Fig 4) to evaluate the effect of live or dead *Candida* administration. Surprisingly, fecal anaerobic bacteria in the genera *Bacteroidales*, *Lachonospiraceae*, *Clostridiales* and *Helicobacter*, but not gram negative bacteria, were increased after live- or heat-killed fungi administration. In parallel, bacteria in the genus of *Lactobacillus* (Fermicutes group) were slightly decreased compared with control mice. *Bacteroides fragilis* is the most common anaerobic bacteria isolated from intra-abdominal sepsis either in patients [23] or mice [24] and *Lactobacilli* help in controlling gut-pathogenic bacteria [25]. Because of the slow progressive clinical characteristics of anaerobic bacterial infection [26], the alteration of the distribution and prevalence of the gut microbiota components did not explain the rapid progressive sepsis in CLP with *Candida* administration.

### Purified (1→3)-β-D-glucan (Pachyman) with heat-killed *E*. *coli* preparation, synergistically increased macrophage cytokine production

Due to the importance of macrophages in sepsis-pathogenesis [27], the immune responses of bone marrow-derived macrophages were tested *in vitro*. Purified (1→3)-β-D-glucan (Pachyman) and heat-killed *E*. *coli* preparation have previously been as representative of Pathogen Associated Molecular Patterns of fungi (19) and bacteria, respectively. These were utilized to assess macrophage responses to them individually and together.

**Fig 5:**
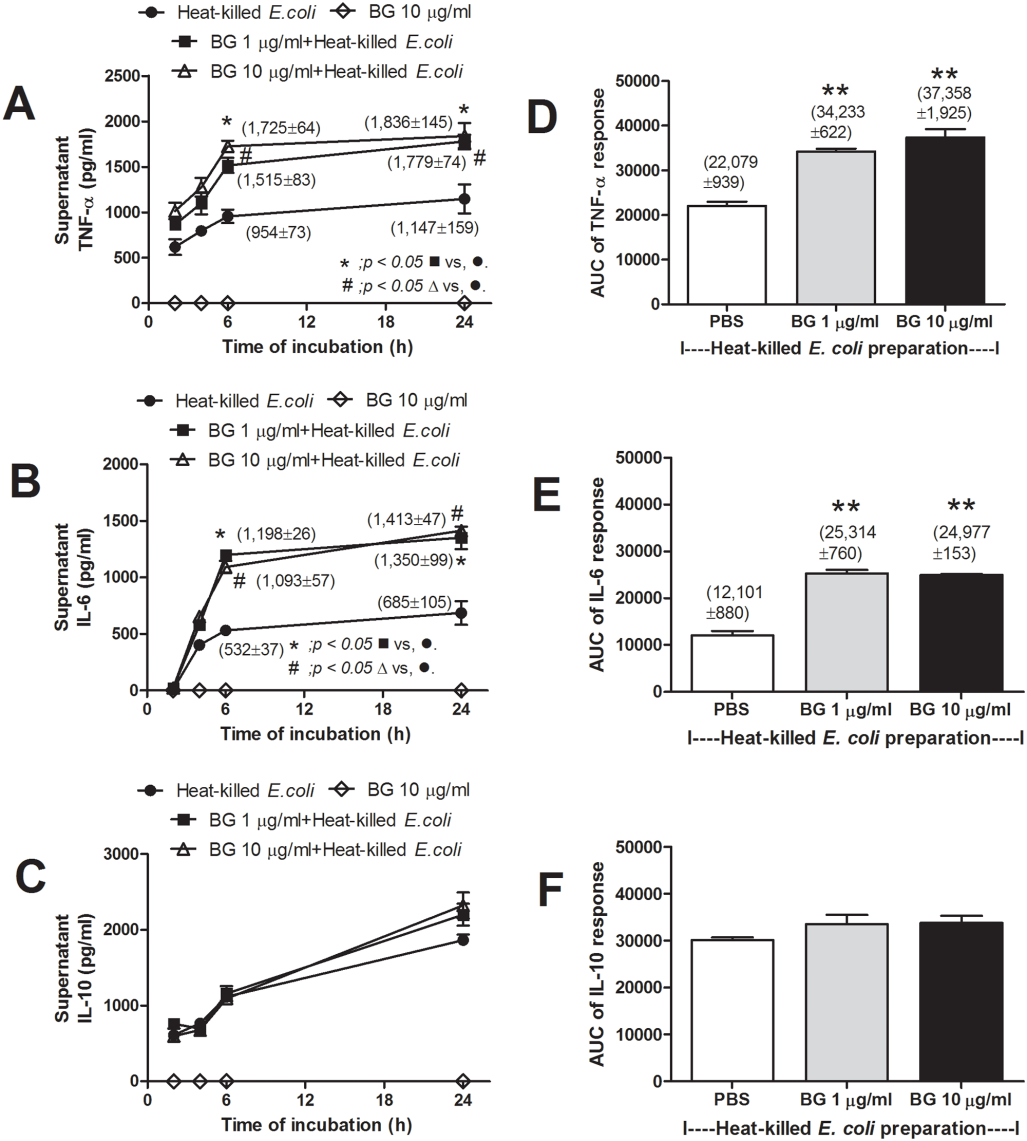
Time-course of cytokines in supernatant of macrophages after incubation with heat-killed *E*. *coli* preparation (see Method) with or without purified (1→3)-β-D-glucan (Pachyman) (BG) (at 1 or 10 μg/ml) or BG 10 μg/ml alone was shown (independent experiments were done in triplicate) (A-C). Area under the curve of cytokine responses of graph A-C are demonstrated (D-F). **, *p* < 0.05 vs. PBS+heat-killed *E*. *coli*.

BG alone, either at 1 or 10 μg/ml could not activate cytokine production from macrophages (Fig 5A–5C; only BG at 10 μg/ml was shown). However, incubation of macrophages with BG plus heat-killed *E*. *coli* resulted in higher levels of TNF-α and IL-6, but not IL-10, and as early as 6h after incubation (Fig 5A–5C). The area under the curve (AUC) of cytokine responses from the data in time-points supported the synergistic effect of BG and *E*. *coli* in TNF-α and IL-6 elicitation but not IL-10 (Fig 5D–5F). However, the elicitation synergy was not BG-dose dependent, above 1 μg/ml.

## Discussion

A positive impact of human commensal intestinal fungi upon murine bacterial sepsis, in the absence of fungemia, was demonstrated. Indeed, oral-administration of *C*. *albicans* (live- and heat-killed) prior to CLP increased serum BG and enhanced sepsis severity. Interestingly, fluconazole reduced intestinal fungi and attenuated sepsis severity. Gut-translocation of BG, and likely other PAMPs, from commensal fungi appeared to enhance sepsis severity, possibly through synergistic stimulation of pro-inflammatory cytokine production by macrophages.

Commensal organisms are necessary for the host immune maturation and gut barrier function [28] but they are also capable of inflammatory response activation [29]. As such, excess probiotic administration activates inflammatory responses and causes inflammatory diarrhea [30, 31]. Because overgrowth of *C*. *albicans*, the predominant gut commensal fungi of human, is observed with antibiotic administration [32, 33], significant *Candida* colonization is presumed in patients with sepsis. Interestingly, the “*Candida* colonization index” is an indicator for candidiasis prediction in patients with sepsis but not the index for bacterial sepsis severity [34, 35]. However, the potential exacerbation of bacterial sepsis due to intestinal *Candida* has not been previously explored. Accordingly, *Candida* oral-administration in a dose that was adequate for the induction of fecal *Candida*, without the production of candidemia, was selected to evaluate its effects in a bacterial sepsis model.

Although CLP with oral administration of *C*. *albicans* at highly elevated dose resulted in candidemia, *C*. *albicans* dosing at 10^6^ CFU induced positive fecal fungi culture without candidemia and was selected for use in our experiments. Live- or heat-killed- *Candida* administered prior to CLP produced more elevated serum BG and more severe sepsis. Sepsis alone induced elevated serum BG and *Candida*-administration, with sepsis, showed further increases in the BG level. Additionally, CLP with heat-killed *Candida* induced higher serum BG than CLP alone. Further, fungal recovery from the mesenteric lymph node, an indicator of gut-translocation, was negative. These data imply the importance of gut-translocation of fungal molecules but, perhaps, not viable cells. Moreover, fluconazole reduced fecal fungal burdens, serum BG and attenuated sepsis severity in CLP with live-*Candida* administration but not CLP-alone. This supports the potential importance of intestinal fungi as a source of serum BG in bacterial sepsis.

Another factor that could be responsible for the more severe sepsis after an oral fungi administration is the alteration in gut microbiota. As such, microbiota analysis showed the increase in *Bacteroidales*, *Lachonospiraceae*, *Clostridiales* and *Helicobacter* but decreased *Lactobacillus* group after fungi administration. Although bacteremia from these bacteria is not frequently found or presents with limited severity [23], *Bacteroidales* bacteremia is commonly demonstrated in patients, with relatively high virulence, such as with *Bacteroides fragillis* infection [36]. The *Bacteroidales* are also the predominant component of the bacterial microbiota in human intestine. It is also generally observed that *E*. *coli* is the most active cause of infection within 24h after intra-abdominal injury. In addition, anaerobic bacteria induce a chronic stage of infection [26]. Hence, *Bacteroides* infection could not explain the rapid progression of sepsis (most of the mice died within 24h) of CLP with *Candida* administration. On the other hand, *Lactobacillus* spp., which are helpful components of the intestinal microbiota for the attenuation of gut leakage [25], were decreased after fungi administration. We recently demonstrated the attenuation of gut leakage with *Lactobacilli* in a *C*. *difficile*-induced sepsis model [37]. Thus, decreased *Lactobacilli* might enhance gut-leakage in our model resulting in the translocation of gut BG.

Although the increased sepsis severity in our models could be responsible from fungal factors (fecal *Candida* burdens and serum BG) and/or bacterial factor (gut microbiome alteration), the data from the CLP-*Candida* model that i) the oral administration of live- or heat killed- *Candida* increased sepsis severity and ii) the reduction of fecal *Candida* with fluconazole, without additional antibacterial drugs, attenuated sepsis severity implied a more prominent influence of fungal factors over bacterial factor in bacterial sepsis severity in our models. It is also interesting to note that the impact of intestinal *Candida* on bacterial sepsis severity was also more predominant than the effect from fecal-microbiome alteration in the 5 days *Candida* colonization model, a model with the influence of mixed-oral antibiotics [14].

Because serum BG in normal mice is usually absent or very low (< 30 pg/ml), it is interesting to note that serum BG elevation is demonstrated after CLP without *Candida* administration (Fig 2E). Without *Candida* introduction, measurement of BG in gut contents was already at the highest value of the assay (> 523 pg/ml). Thus the intestinal contents contribution of BG is due to either foodstuffs or un-culturable fungi. However, *Candida* administration increases gut BG as indirectly demonstrated by the higher serum BG in *Candida*-CLP compared to CLP alone. Taken together, *Candida* administration possibly increases pathogenic anaerobes, decreases beneficial bacteria, and enhances gut translocation of BG (from foodstuffs and/ or gut-fungi). These *Candida*-driven factors may be responsible for the exacerbation of sepsis. In the late phase of sepsis, serum BG reduced in survivor mice at 96h post-CLP but not reach the level of the sham control, in contrast to serum cytokines that already normalized at 96h post-CLP. The compatibility between serum BG and serum cytokines in the early phase of sepsis (18h post-CLP) and the discordance of these parameters in the late phase of sepsis (96h post-CLP) implies the different influence of serum BG in the different phase of sepsis. More studies in this topic are needed. The incubation of BG with heat-killed *E*. *coli* preparations enhanced pro-inflammatory cytokine production from macrophages in comparison with *E*. *coli* preparation alone. Although there was no direct *in vivo* data from our models, the *in vitro* macrophage characteristic implied the alteration of macrophage functions through dual-stimulation by both bacterial and fungal molecules [20, 38, 39]. All in all, the potential importance of fecal *Candida* to bacterial sepsis, which has been overlooked, was demonstrated. Our data also supported the potential usefulness of serum BG for determining propensity to, or severity of, sepsis.

Translationally, an evaluation of fungal abundance in patient stool and the evaluation of serum BG might be useful in the characterization of sepsis severity. However, stool collection in critical care conditions is labor intensive and potentiates contamination. Hence, the rapid estimation of intestinal contents translocation using a surrogate marker such as serum BG may be appropriate in clinical practice. Because serum BG correlated with intestinal fungal-abundance in sepsis (the present data), associated with impaired gut-permeability barrier [7], and correlated with bacterial sepsis severity [14], we propose the detection of serum BG, but not fecal fungi, as an additional biomarker for sepsis severity. Whether serial characterization of serum BG titers can be useful in the management of septic patients will require significant additional clinical study.

In conclusion, increased intestinal burdens of fungal material, live or dead, was shown to cause increased sepsis severity, in a murine CLP model. This was associated, at least in part, with translocation of fungal material from the gut lumen, enhancing cytokine production and inflammation.

## Supporting information

S1 File “NC3Rs ARRIVE guidelines checklist (fillable).pdf”. (PDF)
